# *Thiobacillus denitrificans* enhances nitrogen removal, growth performance, gut histomorphometry, and immunity in fish raised in recirculatory aquaculture systems

**DOI:** 10.3389/fphys.2026.1963898

**Published:** 2026-09-09

**Authors:** Zonghui Chen, Xiaoyan Jiang, Chengwen Sun, Chunhua Liu, Zhijun Liu, Changchen Zhao

**Affiliations:** Pearl River Fisheries Research Institute, Chinese Academy of Fishery Sciences, Guangzhou, China

**Keywords:** growth performance, Nile tilapia, nitrogen removal, recirculatory aquaculture system, *Thiobacillus denitrificans*, water quality

## Abstract

Recirculatory aquaculture systems (RAS) are one of the most sustainable solutions for Nile tilapia, *Oreochromis niloticus*, production. Nitrogenous waste accumulation has been a major constraint to its usage, especially on water quality and fish health. Effects of *Thiobacillus denitrificans* on nitrogen removal, water quality, microbial abundance, and growth performance as well as the immunity of Nile tilapia cultured in RAS were examined in this study. Three treatments were prepared that comprised a control (CTR = no inoculation, standard biofiltration, and sulfur pellets as electron donor), a conventional nitrifying biofilter (CNB = enriched with *Nitrosomonas* and *Nitrobacter* (1.0 × 10^6^ CFU mL^-1^)), and a *T. denitrificans*-inoculated biofilter (TDB = enriched with *T. denitrificans* (1.0 × 10^6^ CFU mL^-1^) for 56 days). Results showed that total ammonia nitrogen (*P* = 0.001), nitrite (*P* = 0.001), and nitrate (*P* = 0.001) were greatly lowered in TDB compared to the CTR, with the lowest levels found in TDB and the highest levels found in CTR. Also, total suspended solids (*P* = 0.001) and chemical oxygen demand (*P* = 0.001) were significantly lower in TDB than in the CTR group. It is observed that fish on TDB had a considerably increased final body weight (+44.4%) and body weight gain (+49.2%) compared to the control. The addition of TDB in RAS used to raise Nile tilapia greatly (*P* = 0.001), increasing TP level accompanied by marked reduction in GLU (*P* = 0.001) and CHO (*P* = 0.024) and ALT (*P* = 0.021) levels. Antioxidant profiles, acetic viscosity, and butyric acid (*P* < 0.05) were significantly elevated in fish raised in RAS inoculated with the respective microbial treatments compared to the fish in the control group, as was gut histomorphometry. Also, immune profiles (respiratory burst activity, lysozymes, protease, and esterase activities) were significantly (*P* < 0.05) elevated in fish raised in TDB, while the least were observed in the control group. This study demonstrated that *T. denitrificans* improves nitrogen cycling and removal; water stability in RAS, which results in indirectly induced growth; and enhanced immunity in Nile tilapia, thus offering a promising biotechnological approach for sustainable aquaculture.

## 1 Introduction

Aquaculture is contributing more than 50% of global fish production to meet fish food demand (FAO, 2024). Recirculatory aquaculture systems (RAS) are progressively encouraged for sustainable fish farming because they reduce the use of water, improve biosecurity, and are intensive farming system in addition to high yield within a relatively small area. In addition, the controlled culture environment and improved biosecurity of RAS provide greater opportunities for managing environmental conditions and maintaining stable production. These characteristics are particularly important for intensive Nile tilapia production, where increasing stocking densities and feed inputs inevitably increase the generation of nitrogenous wastes. The sustainability advantage of RAS, however, depends on the efficiency of its water-treatment processes because the same water is repeatedly returned to the culture tanks.

Tilapia is the third most farmed finfish in inland aquaculture with a yield up to 4.4 million metric tonnes globally (FAO, 2022). Tilapia farming techniques vary across country due to business opportunities, technical know-how, and availability of resources, including feed and fish species (FAO, 2021). Nile tilapia, *Oreochromis niloticus*, a fish of interest to many farmers, is produced intensively because of its high growth rate and market values leading to high productivity and profitability (El-Sayed, 2019). In spite of the success recording in tilapia production, the sector has been hindered by nitrogenous waste resulting in high mortality and bankruptcy.

However, nitrogenous waste generation and accumulation have become a major challenge in aquaculture. Ammonia (NH_3_) levels in the culturing water are from fish excretion and uneaten feed, which, through nitrification, are oxidised to nitrite (NO_2_^-^) and nitrate (NO_3_^-^). If continuously accumulated, nitrate induces chronic stress, immunosuppression, and growth depression in fish, especially in a closed system like RAS (Luo et al., 2022). Generally, management of RAS is usually through the use of biofilters containing nitrifying bacteria such as *Nitrosomonas* and *Nitrobacter* species, which is effective in ammonia oxidation; however, a complete nitrogen removal is hardly achieved. A major limitation of RAS is therefore the progressive accumulation of dissolved nitrogenous metabolites. Fish excrete ammonia directly into the water, while uneaten feed and fecal material contribute additional nitrogen (Luo et al., 2022). Nitrogen is remained in the recirculating water predominantly as nitrate and could progressively accumulate when water exchange is limited. Although nitrate is generally less acutely toxic than ammonia and nitrite, prolonged exposure to elevated nitrate concentrations can impose chronic physiological stress, impair growth, compromise immune function, and reduce the overall health and performance of cultured fish (Luo et al., 2022). This problem is particularly relevant to RAS because the high degree of water reuse may allow nitrate to accumulate over time if it is not biologically removed. Therefore, there is need for an alternative solution to close the nitrogen cycle within RAS.

*Thiobacillus denitrificans* is an autotrophic bacterium that is capable of reducing sulfur compounds such as thiosulphate, resulting in reducing nitrate to dinitrogen gas. Moreover, *T. denitrificans* is able to grow under low organic carbon conditions due to its high metabolic activities, and hence is used in aquaculture biofilters (Wei et al., 2021). Martínez-Espinosa (2020) described the efficiency of sulphur-driven denitrification in wetlands and wastewater treatment, but its application in RAS aquaculture is underexplored. Studies have shown that sulfur-driven autotrophic denitrification has been extensively used to remove nitrate in wastewater, groundwater, and leachate treatment, its application in RAS needs careful consideration because sulfur oxidation can consume alkalinity and potentially reduce pH, thereby compromising the water-quality required for fish production. Previous studies have also investigated sulfur-based and combined iron–sulfur denitrification in aquaculture systems (Christianson et al., 2015; Ma et al., 2024). However, the effectiveness of a denitrifying microbial inoculant in enhancing sulfur-driven nitrogen removal while simultaneously maintaining water quality and improving fish physiological performance remains insufficiently understood. In particular, *Thiobacillus denitrificans*, a chemolithoautotrophic sulfur-oxidizing denitrifier capable of reducing nitrate to dinitrogen gas under low organic-carbon conditions, may provide an opportunity to strengthen biological denitrification within RAS biofilters.

To the best of our knowledge, no study has holistically demonstrated the integration of the chemolithoautotrophic denitrifier, *T. denitrificans*, into RAS for Nile tilapia production to simultaneously explore the reduction of nitrogenous wastes and organic load. Accordingly, incorporating *T. denitrificans* into RAS could provide a means of coupling nitrification and denitrification, thereby preventing nitrate accumulation, improving water quality, reducing the need for water exchange, and ultimately supporting fish growth and health. Also, there is a need to link biofilter-mediated water quality parameters to fish growth and immunity. Therefore, integration of *T. denitrificans* into RAS could enhance nitrogen removal, improve water quality, and boost fish growth. Thus, this study examined the effects of *T. denitrificans* on nitrate concentrations, microbial abundance, and water quality in Nile tilapia raised in RAS.

## 2 Materials and methods

### 2.1 Ethical approval

The protocol of the project was approved by the Animal Use Ethics Committee of the Pearl River Fisheries Research Institute, Chinese Academy of Fishery Sciences, Guangzhou, Guangdong, China (2024-002115). This study was performed in line with the principles of the Declaration of Helsinki. Approval was granted by the Ethics Committee (Animal Care Use and Research Ethics Committee) of the Pearl River Fisheries Research Institute, Chinese Academy of Fishery Sciences, Guangzhou, Guangdong, ACUREC- (2024-002115) China.

### 2.2 Assessment of fish health

Fish used for this study were purchased from the laboratory of the Fisheries Research Institute, Chinese Academy of Fishery Sciences, Guangzhou, Guangdong, China. The fish were screened for any clinical symptoms of infections, swimming behaviour, and response to feed (Adeshina et al., 2025). Briefly, morphological assessments such as skin, eye, and gills colour were examined as well as mucus secretion levels. In addition, fish were quarantined in a salt solution of 2 NaCl g/L for 24 h (American Veterinary Medical Foundation, 2020).

### 2.3 Acquisition and preparation of *T. dinitrificans* isolates

*T. dinitrificans* (ATCC 25259) was purchased from Chromachemie Laboratory, India, and was reconstituted into Nutrient Agar (NA) at 35 °C and prepared in NA broth to contain 1.0 × 10^6^ CFU mL^-1^. The concentration was based on the manufacturer’s recommendation as an effective dosage to effectively establish biofilms, compete with indigenous microbes, and remove nitrogenous wastes without causing oxygen depletion or disrupting the balance of nitrifiers (Beller et al., 2006; Zhang et al., 2021; Wang et al., 2022; Asforooshani et al., 2025).

### 2.4 Fish culture and biofilter inoculation

Nile tilapia fingerlings (n = 300; mean weight = 3.0 ± 0.2 g) were acclimatized for four weeks and fed a commercial diet (30% crude protein). Then 180 fish (15.4 ± 0.3 g mean ± standard deviation) were randomly allotted to nine aquaria of 100-L capacity (dimension: 80 cm × 40 c × 32 cm; length × width × depth) connected to nine RAS, aerated, and operated at a 12:12 h photoperiod, while the remaining fish were returned to the farm. Each aquarium contained 20 fish, comprising three treatments with three replicates. The choice of this size is due to the early exponential growth stage and response to the feed/treatments and environmental conditions. Fish were fed with 30% crude protein commercial feed four times per day, specifically at 8:00, 11:00, 14:00, and 18:00 h until apparent satiation for 56 days. The aquaria were siphoned daily to avoid wastes particles generated from feces and uneaten feed. The experimental treatments consist of control (CTR = no inoculation, standard biofiltration, and sulfur pellets as electron donor), conventional nitrifying biofilter (CNB = enriched with *Nitrosomonas* and *Nitrobacter* (1.0 × 10^6^ CFU mL^-1^) at a ratio of two to one, respectively, and sulfur pellets as electron donor), and *T. denitrificans*-inoculated biofilter (TDB = enriched with *T. denitrificans* (1.0 × 10^6^ CFU mL^-1^) and sulfur pellets as electron donor). The sulfur pellets in powder form (Solution Healthcare Services LLC, USA) were applied at 1 g/L, using an effective particle size of approximately 0.30 mm. This level was selected based on recommendation of Christianson et al., 2015 to provide sufficient electron-donor availability for denitrification while minimizing excessive sulfur oxidation and associated reductions in alkalinity and pH. The same sulfur-pellet level and form were maintained across the relevant treatments to ensure that sulfur availability was not a confounding factor and that differences in nitrogen removal could be attributed primarily to *T. denitrificans* inoculation. Standard biofiltration herein refers to the naturally established microbial biofilter community responsible for routine biological transformation of nitrogenous wastes. Biofilters were preconditioned for 10 days before stocking fish.

### 2.5 Evaluation of water quality parameters

The water quality parameters were inspected on a regular basis after every 24 h. For this, dissolved oxygen (DO), alkalinity, and temperature were measured with the aid of a dual-parameter EcoSense meter (YSI, Model No. DO200A, China) while pH was measured using a digital pH meter (Model Photoic 20, Labtech International Ltd.). Ammonia (Cat. No.: Cod. 9162-5), nitrate, and nitrite (Cat. No.: COD. 2005-2) were measured using diagnostic kits from LabconTest Aqua Doce, Brazil. Chemical oxygen demand (COD) (Cat. No.: A-7301) was measured using CHEMetrics Aquaphonix Scientific, USA, while total suspended solids (TSS) were determined with the aid of a TSS kit (Cat. No.: LXV322.99.00002, HACH, USA) (APHA, 2022).

### 2.6 Microbial community analysis

Biofilm samples from biofilters were swabbed at days 0, 30, and 56 and released into sterile water for 3 h. One mL from each sample was serially diluted at a dilution factor of four. From each sample, one mL was dispensed into two separate petri dishes to receive plate count agar (PCA) for total bacteria count (LAB M, LAB149) and MacConkey agar (LAB M, LAB002) for total coliform count using the pure plate count method (Adeshina et al., 2016). The media swirled when cooled to 50 °C, then allowed to solidified before incubation (Newlife Laboratory Incubator NL-9052-1) for 24 h at 37 °C in triplicates. The formed distinguished colonies to form were counted on a Wincom colony counter (16 W, 220 V ± 10%, 50 Hz) and expressed in Log_10_CFU mL^-1^ (Adeshina et al., 2016).

### 2.7 Fish growth evaluation

At the end of the culturing period, the fish were starved for 24 h and anesthetized with 200 mg L^-1^ of tricaine methane-sulfonate (MS-222, Syndel, Ferndale, Washington, USA) (Adeshina et al., 2025) before being group-weighed. Growth parameters were then calculated as follows:

Body weight gain (BWG, g) = final body weight (FBW, g) – initial body weight (IBW, g);
Daily body weight gain (DBWG, g/day) = BWG (g)/experimental period;
Specific growth rate (SGR; % day^-1^) = 100 [Ln FBW (g) − Ln IBW (g)]/Duration of trial;
Feed intake (FI) = total feed distributed/number of fish
Feed conversion ratio (FCR) = FI (g)/BWG (g);
Fish survival (FS) = 100 (final number of fish/initial number of fish).

### 2.8 Sample collection

Randomly, four fish from each aquarium were selected and anesthetized for 2 minutes in 30 mg/L of sodium bicarbonate buffered with tricaine methane sulfonate (MS222, 30 mg/L, Syndel, Ferdale, Washington, USA) (American Veterinary Medical Foundation, 2020). From which 5 mL of blood was collected from the caudal vein of these fish for hematology and plasma analysis. The blood was divided into two units. The first part was kept in anticoagulant bottles containing 20 U/L lithium heparin at room temperature to measure hematological profiles. The second unit of the blood samples was centrifuged (Model: LC400, Joanlab Laboratories, China) at 8,000 × *g* for 10 minutes to prepare the plasma and stored at -80 °C. The fish were then euthanized in 800 mg/L MS-222 (Syndel, Ferndale, Washington, USA) (American Veterinary Medical Foundation, 2020). In addition, samples of intestinal content were extracted into a 1,000 µL micropipette, frozen in liquid nitrogen, and stored at -80 °C for short-chain fatty acids (SCFAs) and gut microbial content analyses, while 2-cm gut samples were collected for intestinal histomorphometry evaluation. The remaining parts of the fish were discarded according to American Veterinary Medical Foundation (2020).

### 2.9 Evaluation of haematological profiles

The packed cell volume (PCV, %), red blood cells (RBCs, × 10^6^ µL^-1^), white blood cells (WBC, × 10³ µL^-1^), and platelets (PLT, × 10^6^ µL^-1^) were determined according to Van Kampen and Zijlstra (1961), while haemoglobin (Hb, g dL^-1^) was measured using the Brown (1980) method. In addition, using the Wright-Giemsa stain method, differential counts, i.e., lymphocytes (LYM, %), heterocytes (HET, %), monocytes (MON, %), eosinophils (EOS, %), and basophils (BAS, %) were estimated.

### 2.10 Plasma biochemical and liver enzyme parameters

From the plasma samples, total protein (TP, g dL^-1^; Cat. No. TP3869) concentration, glucose (GLU, mg dL^-1^; Cat. No. 3815), and total cholesterol (CHO, mg dL^-1^; Cat. No. CH3810) were evaluated with the aid of colorimetric tests (Randox^®^ Laboratories Ltd, Crumlin, County Antrim, United Kingdom). Aspartate aminotransferase (AST, IU mL^-1^) (Cat. No. AS8005), alkaline phosphatase (ALP, IU mL^-1^) (Cat. No. AP307), and alanine aminotransferase (ALT, IU mL^-1^) (Cat. No. AL3801) were measured using the colorimetric method (Randox^®^ Laboratories Ltd, Crumlin, County Antrim, United Kingdom) (da Cruz et al., 2024).

### 2.11 Assessment of antioxidant profiles

Antioxidant profiles of the fish were evaluated using diagnostic kits from Invitrogen™ ThermoFisher Scientific Inc. For superoxide dismutase (SOD, IU mL^-1^), catalase (CAT, IU mL^-1^), and concentration of malondialdehyde (MDA, nmol L^-1^) were analysed using assay kits with Cat. No. EIASODC, Cat. No. EIACATC, and Cat. No. EEA015 for SOD, CAT, and MDA, respectively (McCord and Fridovich, 1969; Aebi, 1984).

### 2.12 Short-chain fatty acids

The method of Bianchi et al. (2011) was used to evaluate the Short-chain fatty acids (SCFAs) (acetic (nmol L^-1^), propionic (nmol L^-1^), butyric (nmol L^-1^) acids), and pH levels. Briefly, a solution containing 0.5 g of the sample in 2.5 mL of distilled water and 0.25 g of sodium chloride was centrifuged at 3,586 × *g* for 10 minutes before dispensing 1.5 mL of the supernatant into 20 μL of a 0.9 M H_2_SO_4_ solution (pH 2) and transferring it into a 4.0-mL vial and sealing it with a PTFE/rubber septum. The short-chain fatty acids (carboxen-polydimethylsiloxane) above the fibre (10 mm × 75 μm film thickness, Supelco^®^, Bellefonte, PA, USA) were extracted using the headspace solid-phase microextraction (HS-SPME) technique at 40 °C for 30 minutes. (Mombach et al., 2019) The content was assessed using a gas chromatograph with a flame ionisation detector (GC-FID; Varian Star 3400CX CA, USA) and compared to real standard retention times (Sigma, Sigma-Aldrich Brazil Ltda, São Paulo, Brazil).

### 2.13 Intestinal histomorphometry

Following the euthanasia of fish and intestinal sample collection as described in section 2.5, samples were kept in buffered formalin (20%) from Sigma-Aldrich, St. Louis, MO, before being transferred to Bouin’s fluid (Eyarefe et al., 2008). A dehydration was performed on the tissues in graduated ethanol (70, 80, 90, 95, and 100%) and embedded in paraffin wax. After that, 5 μm sections were cut and dewaxed in xylene and immediately hydrated in ethanol in 100, 95, 90, 80, and 70% order before staining in hematoxylin and eosin (Sigma-Aldrich, St. Louis, MO). The samples were then viewed under an Olympus CX21 light microscope. The villi width (VW, cm), villi length (VL, cm), and cryptal depth (CP, cm) were measured, while the intestinal area of absorption (AA, cm²) was estimated as follows:

Area of absorption (cm²) = VL (cm) × VW (cm).

### 2.14 Determination of immune responses

Respiratory burst activity (RBA, µmol) was measured using the Randox diagnostic reagent kit (Randox, London, UK). In brief, 100 µL of whole-blood suspension was released into each well of a 96-well microtiter plate (MaxiSorp™ 96, Nalge-Nunc, Hereford, UK) following an incubation with 1 mg/mL NBT solution at 37 °C for 15 min. Then, the formazan precipitate dissolved in 5% dimethyl sulfoxide. Absorbance was measured at 570 nm using a microplate reader (MaxiSorp™ 96, Nalge-Nunc, Hereford, UK). The RBA was calculated according to the manufacturer’s instructions. Also, briefly, serum samples were incubated with a suspension of *Micrococcus lysodeikticus* cells as the substrate. The reduction in turbidity resulting from bacterial cell wall lysis was measured spectrophotometrically at 600 nm using a microplate reader (HINOTEK, AMR-100). A standard curve was prepared using lysozyme from chicken egg white (Sigma-Aldrich, Cat. L7651). Lysozyme activity was estimated from the standard curve and expressed as the chicken egg-white lysozyme specific activity of approximately 40,000 U/mg lysozyme (40 U µg^-1^) and total serum protein = 30 mg/mL. Protease activity (PTA, U/mg protein), alkaline phosphatase (APP, IU/L), and esterase activity (ETA, U/mg protein) as described by Adeshina et al. (2021).

### 2.15 Statistical analysis

Data were subjected to Kolmogorov–Smirnov and Bartlett tests for determination of homogeneity of variances and normality of distribution. Then, one-way analysis of variance was used to analyze the data to investigate the influence of *T. denitrificans*, sulfur supplementation, and their interaction for nitrogen removal, general microbial abundance, water quality, and zootechnical parameters of Nile tilapia, while Tukey’s test was performed to separate significantly different means at a significance level of *P < 0.05* with the Social Package for Social Science (SPSS) version 20, Richmond, VA, USA (Dytham, 2011).

## 3 Results

### 3.1 Nitrogen removal

**Table 1** depicted the nitrogen removal in water used to raise Nile tilapia in *T. denitrificans-*inoculated RAS. Nitrogen was significantly (*P* < 0.05) reduced in RAS inoculated with *T. denitrificans* compared to the control. TAN (*P* = 0.001), nitrite (*P* = 0.001), and nitrate (*P* = 0.001) were greatly lowered in TDB compared to the CTR, with the lowest levels found in TDB and the highest levels found in CTR (**Table 1**).

**Table 1:** Nitrogen removal levels in Nile tilapia raised in RAS inoculated with *T. denitrificans* for 56 days.

Parameters (mg L^-1^)	CTR	CNB	TDB	PSD	*P* value
TAN	2.0 ^a^	1.2 ^b^	0.5 ^c^	0.100	0.001
Nitrite	2.0 ^a^	1.0 ^b^	0.2 ^c^	0.020	<0.001
Nitrate	78.3 ^a^	68.5 ^b^	14.7 ^c^	5.524	0.001

Means having the same letter in the same row are not significantly different at *P* > 0.05.

TAN, Total ammonia nitrogen; CTR, no inoculation, standard biofiltration and sulfur pellets as electron donor; CNB, conventional nitrifying biofilter, enriched with *Nitrosomonas* and *Nitrobacter* (1.0 ×10^6^ CFU mL^-1^) at ratio of two to one, respectively and sulfur pellets as electron donor; TDB, *T. denitrificans*-inoculated biofilter, enriched with *T. denitrificans* (1.0 ×10^6^ CFU mL^-1^) and sulfur pellets as electron donor), PSD, Pooled standard deviation.0.

### 3.2 Water quality

Water quality parameters were shown in **Table 2**. Water quality parameters were greatly (*P* < 0.05) influenced by inoculation of *T. denitrificans* in the RAS. Temperature and DO remain relatively stable (*P* > 0.05) across the treatments; pH was higher (*P* = 0.018), while TSS (*P* = 0.001) and COD (*P* = 0.001) were significantly lower in TDB than in the CTR group (**Table 2**).

**Table 2:** Water quality parameters in Nile tilapia raised in RAS inoculated with *T. denitrificans* for 56 days.

Parameters	CTR	CNB	TDB	PSD	*P* value
Temp °C	25.2 ^a^	25.7 ^a^	26.9 ^a^	0.453	0.127
DO (mg L^-1^)	6.1 ^a^	6.2 ^a^	6.2 ^a^	0.302	0.356
pH	7.2 ^b^	7.5 ^a^	7.6 ^a^	0.101	0.018
TSS (mg L^-1^)	42.4 ^a^	29.9 ^b^	24.5 ^c^	5.421	0.001
COD (mg L^-1^)	32.6 ^a^	20.2 ^b^	18.4 ^c^	3.879	0.001

Means having the same letter in the same row are not significantly different at *P* > 0.05.

Temp., Temperature; DO, Dissolved oxygen; pH, Hydrogen ion concentration; TSS, Total suspended solids; COD, CTR, no inoculation, standard biofiltration and sulfur pellets as electron donor; CNB, conventional nitrifying biofilter, enriched with *Nitrosomonas* and *Nitrobacter* (1.0 ×10^6^ CFU mL^-1^) at ratio of two to one, respectively and sulfur pellets as electron donor); TDB, *T. denitrificans*-inoculated biofilter, enriched with *T. denitrificans* (1.0 ×10^6^ CFU mL^-1^) and sulfur pellets as electron donor; PSD, Pooled standard deviation.

### 3.3 General microbial abundance

**Table 3** depicts that the microbial abundance was significantly (*P* < 0.05) different among the treatments. TVC (*P* = 0.002) was significantly higher, while the TEB (*P* = 0.002) was greatly lowered in TDB than in the CTR.

**Table 3:** General microbial abundance in Nile tilapia raised in RAS inoculated with *T. denitrificans* for 56 days.

Parameters (Log_10_CFU/g)	CTR	CNB	TDB	PSD	*P* value
TVC	6.2 ^a^	6.5 ^a^	6.5 ^a^	0.223	0.002
TEB	5.3 ^a^	4.2 ^b^	3.3 ^c^	0.112	0.001

Means having the same letter in the same row are not significantly different at *P* > 0.05.

TVC, Total bacteria count; TEB, Total enterobactericeae; CTR, no inoculation, standard biofiltration and sulfur pellets as electron donor); CNB, conventional nitrifying biofilter, enriched with *Nitrosomonas* and *Nitrobacter* (1.0 ×10^6^ CFU mL^-1^) at ratio of two to one, respectively and sulfur pellets as electron donor); TDB, *T. denitrificans*-inoculated biofilter, enriched with *T. denitrificans* (1.0 ×10^6^ CFU mL^-1^) and sulfur pellets as electron donor), PSD, Pooled standard deviation.

### 3.4 Fish growth and survival

**Table 4** displays Nile tilapia’s growth performance raised in RAS inoculated with *T. denitrificans* for 56 days as well as its fish survival. It is observed that fish on TDB had considerably increased fish growth compared to the control (*P* < 0.05); specifically, FBW (*P* = 0.001), BWG (*P* = 0.001), BDWG (*P* = 0.011), and SGR (*P* = 0.025) were significantly higher in TDB fish in the TDB group than the control. However, the FCR of fish reared in TDB-induced RAS was significantly lower than that which was observed in the control (*P* = 0.005). The TDB inoculation in the RAS did not significantly influence the fish survival (*P* > 0.05) (**Table 4**).

**Table 4:** Growth performance and survival of Nile tilapia raised in RAS inoculated with *T. denitrificans* for 56 days.

Parameters	CTR	CNB	TDB	PSD	*P* value
IBW (g)	15.4 ^a^	15.5 ^a^	15.4 ^a^	0.201	0.153
FBW (g)	158.4 ^c^	172.5 ^b^	228.7 ^a^	7.468	<0.001
BWG (g)	143 ^c^	157 ^b^	213.3 ^a^	2.735	<0.001
BDWG (g/day)	2.6 ^b^	2.8 ^b^	3.8 ^a^	0.443	0.011
SGR (%/day)	4.2 ^b^	4.3 ^b^	4.8 ^a^	0.121	0.025
FCR	1.5 ^a^	1.5 ^a^	0.8 ^b^	0.111	0.005
FI (g)	214.5 ^a^	235.5 ^a^	170.6 ^c^	16.745	<0.001
FS (%)	100.0 ^a^	100.0 ^a^	100.0 ^a^	0.000	0.775

Means having the same letter in the same row are not significantly different at *P* > 0.05.

IBW, Initial body weight; FBW, Final body weight; BWG, Body weight gain; DBWG, Daily body weight gain; SGR, Specific growth rate; FCR, Feed conversion ratio; FI, Feed intake; SR, Fish survival; CTR, no inoculation, standard biofiltration and sulfur pellets as electron donor; CNB, conventional nitrifying biofilter, enriched with *Nitrosomonas* and *Nitrobacter* (1.0 ×10^6^ CFU mL^-1^) at ratio of two to one, respectively and sulfur pellets as electron donor); TDB, *T. denitrificans*-inoculated biofilter, enriched with *T. denitrificans* (1.0 ×10^6^ CFU mL^-1^) and sulfur pellets as electron donor).

### 3.5 Hematological profiles

The study showed hematological profiles of Nile tilapia cultured with *T. denitrificans* inoculated in RAS were significantly altered (*P* < 0.05; **Table 5**). The PCV (*P* = 0.001), Hb (*P* = 0.001), RBC (*P* = 0.001), WBC (*P* = 0.001), and PLT (*P* = 0.001) were statistically different among the fish. Their highest values were recorded in fish reared in TDB, while the lowest corresponding values were obtained in fish raised in the control group. However, *T. denitrificans* did not significantly alter the differential cell count of HET, MON, EOS, and EOS (*P* > 0.05; **Table 5**).

**Table 5:** Hematological profiles of Nile tilapia reared in *T. denitrificans-*based RAS for 56 days.

Parameters	CTR	CNB	TDB	PSD	*P*-value
PCV (%)	16.70 ^b^	21.33 ^a^	21.43 ^a^	0.616	<0.001
Hb (g/dL)	4.63 ^c^	6.37 ^b^	6.50 ^a^	0.233	<0.001
RBC (x10^6^/μL)	1.23 ^b^	1.53 ^a^	1.53 ^a^	0.068	<0.001
WBC (x10^3^/μL)	149.23 ^b^	186.83 ^a^	191.30 ^a^	0.068	<0.001
PLT (x10^6^/μL)	166.03 ^c^	200.30 ^b^	221.00 ^a^	11.053	<0.001
LYM (%)	51.67 ^b^	57.33 ^a^	58.67 ^a^	1.125	0.050
HET (%)	42.57 ^a^	34.67 ^a^	37.00 ^a^	1.167	0.069
MON (%)	3.00 ^a^	3.00 ^a^	3.00 ^a^	0.364	0.165
EOS (%)	2.67 ^a^	3.00 ^a^	3.17 ^a^	0.316	0.674
BAS (%)	0.67 ^a^	0.67 ^a^	1.00 ^a^	0.163	0.275

Means having the same letter in the same row are not significantly different at *P* > 0.05.

PCV, Packed cell volumes (%); Hb, Hemoglobin (g/dL); RBC, Red blood cell (x10^6^/μL); WBC, White blood cell (x10^3^/μL); PLT, Platelets (x 10^3^/μL); LYM, Lymphocytes (%); HET, Heterocytes (%); MON, Monocytes (%); EOS, Eosinophils (%); BAS, Basophils (%); CTR, no inoculation, standard biofiltration and sulfur pellets as electron donor; CNB, conventional nitrifying biofilter, enriched with *Nitrosomonas* and *Nitrobacter* (1.0 ×10^6^ CFU mL^-1^) at ratio of two to one, respectively and sulfur pellets as electron donor; TDB, *T. denitrificans*-inoculated biofilter, enriched with *T. denitrificans* (1.0 ×10^6^ CFU mL^-1^) and sulfur pellets as electron donor; PSD, Pooled standard deviation.

### 3.6 Plasma biochemical and liver enzyme profiles

The addition of TDB in RAS used to raise Nile tilapia greatly increased TP (*P* = 0.001) levels, accompanied by marked reduction in GLU (*P* = 0.001) and CHO (*P* = 0.024) levels (**Table 6**). Regarding the liver enzyme activity of the Nile tilapia TDB group, ALT (*P* = 0.021) level was significantly reduced, and their maximum values were detected in fish in the control system with their corresponding lowest values obtained in the TDB group (*P* > 0.05; **Table 6**). However, AST and ALP levels were not significantly affected among the treatments (*P* < 0.05; **Table 6**).

**Table 6:** Plasma biochemical and liver enzymes profiles of Nile tilapia reared in *T. denitrificans-*based RAS for 56 days.

Parameters	CTR	CNB	TDB	PSD	*P*-value
TP (g/dL)	4.53 ^c^	6.21 ^b^	7.86 ^a^	0.217	0.001
GLU (mg dL^-1^)	142.43 ^a^	135.35 ^b^	117.61 ^c^	6.239	<0.001
CHO (mg dL^-1^)	126.46 ^a^	121.28 ^b^	108.48 ^c^	19.764	0.024
ALT (IU L^-1^)	31.88 ^a^	29.93 ^b^	27.78 ^b^	1.290	0.021
AST (IU L^-1^)	44.20 ^a^	44.11 ^a^	41.95 ^a^	2.965	0.175
ALP (IU L^-1^)	33.55 ^a^	32.58 ^a^	32.18 ^a^	3.453	0.083

Means having the same letter in the same row are not significantly different at *P* > 0.05.

TP, Total protein; GLU, Glucose; CHO, Total cholesterol; AST, Aspartate aminotransferase; ALP, Alkaline phosphatase; ALT, Alanine aminotransferase; CTR, no inoculation, standard biofiltration and sulfur pellets as electron donor; CNB, conventional nitrifying biofilter, enriched with *Nitrosomonas* and *Nitrobacter* (1.0 ×10^6^ CFU mL^-1^) at ratio of two to one, respectively and sulfur pellets as electron donor; TDB, *T. denitrificans*-inoculated biofilter, enriched with *T. denitrificans* (1.0 ×10^6^ CFU mL^-1^) and sulfur pellets as electron donor; PSD, Pooled standard deviation.

### 3.7 Antioxidant activities

**Figure 1:**
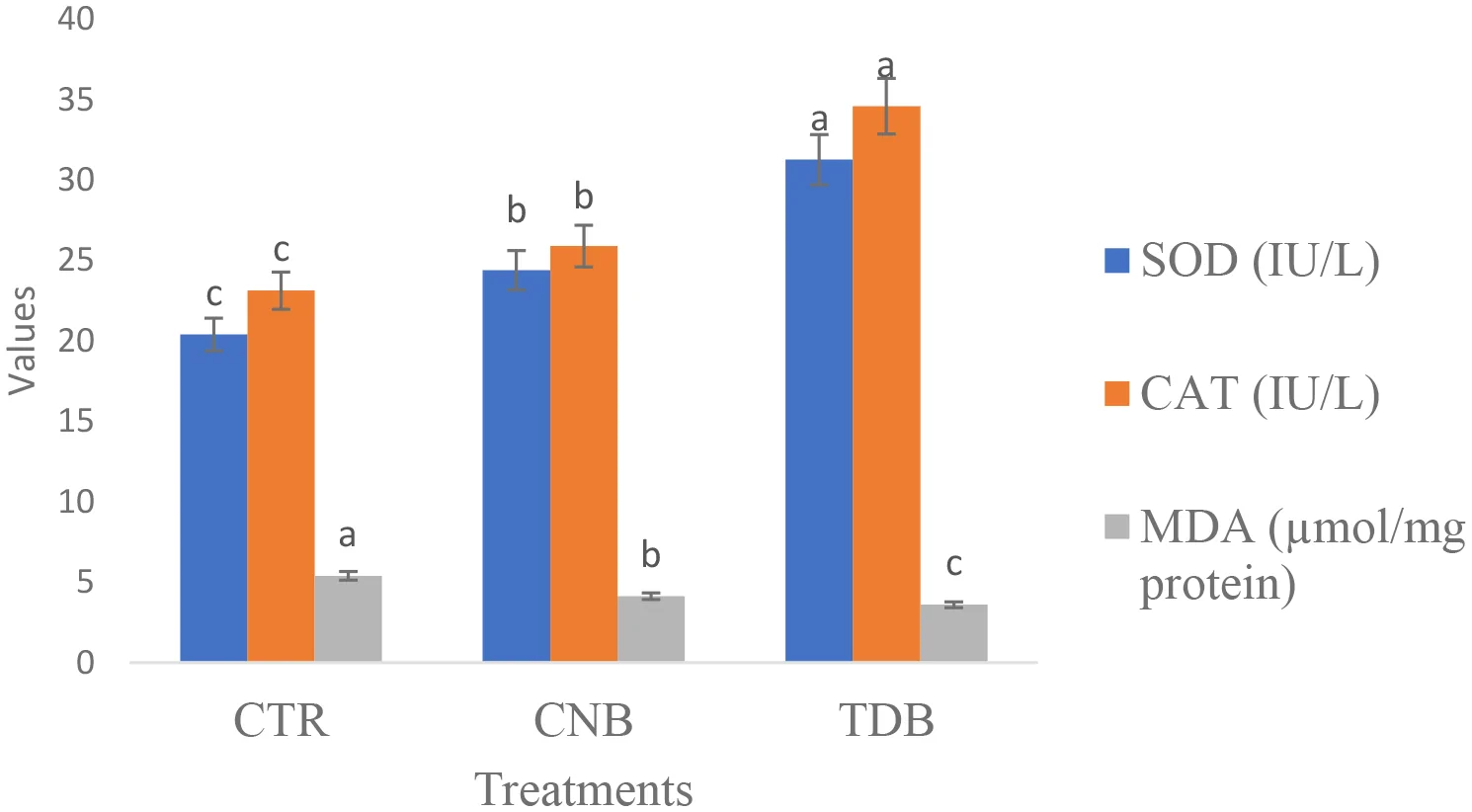
Antioxidant profiles of Nile tilapia raised in RAS for 56 days. Bars of the same colour having the same letter in the same row are not significantly different at *P* > 0.05. Treatments: CTR, no inoculation, standard biofiltration and sulfur pellets as electron donor; CNB, conventional nitrifying biofilter, enriched with *Nitrosomonas* and *Nitrobacter* (1.0 ×10^6^ CFU mL^-1^) at ratio of two to one, respectively and sulfur pellets as electron donor; TDB, *T. denitrificans*-inoculated biofilter, enriched with *T. denitrificans* (1.0 ×10^6^ CFU mL^-1^) and sulfur pellets as electron donor; PSD, Pooled standard deviation.

Antioxidant profiles of Nile tilapia in this study showed significant (*P* < 0.05; **Figure 1**) improvements in TDB compared to the control group. On the other hand, reduction in the levels of MDA (*P* < 0.05) was caused by *T. denitrificans* inoculation. Fish in the TDB system had the lowest MDA level, while the highest was observed in the control (**Figure 1**).

### 3.8 Evaluation of digesta pH, viscosity, and short-chain fatty acids

**Figure 2:**
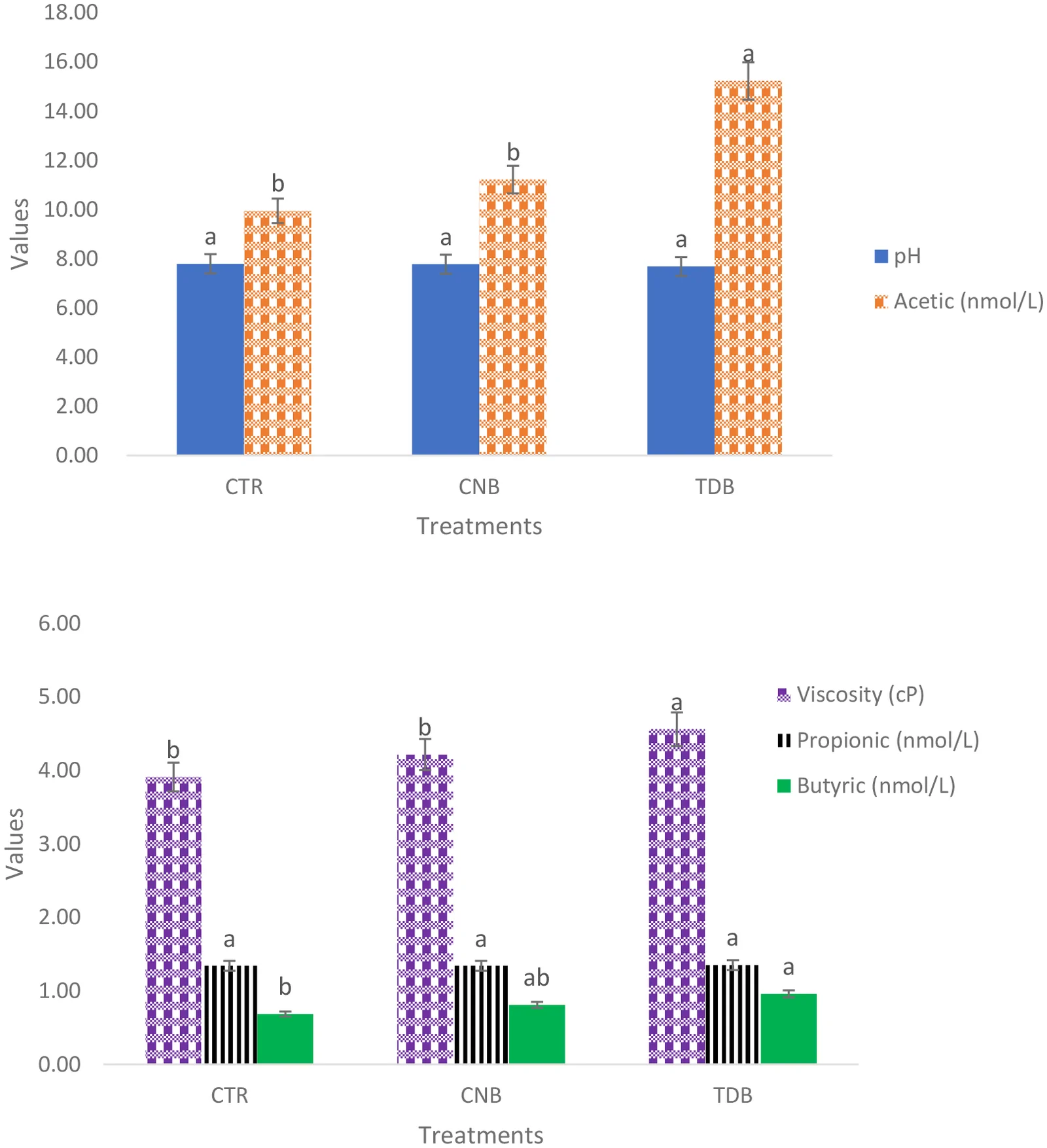
Digesta pH, viscosity, and short-chain fatty acids of Nile tilapia raised in RAS for 56 days. Bars of the same colour having the same letter in the same row are not significantly different at *P* > 0.05. Treatments: CTR, no inoculation, standard biofiltration and sulfur pellets as electron donor; CNB, conventional nitrifying biofilter, enriched with *Nitrosomonas* and *Nitrobacter* (1.0 ×10^6^ CFU mL^-1^) at ratio of two to one, respectively and sulfur pellets as electron donor), TDB, *T. denitrificans*-inoculated biofilter, enriched with *T. denitrificans* (1.0 ×10^6^ CFU mL^-1^) and sulfur pellets as electron donor; PSD, Pooled standard deviation.

**Figure 2** reveals the acetic and butyric acids were significantly elevated (*P* < 0.05) in fish raised in an induced RAS compared to the fish in the control. Conversely, *T. denitrificans* had no significant (*P >* 0.05) influence on the pH and propionic acid (**Figure 2**).

### 3.9 Gut histomorphometry

**Figure 3:**
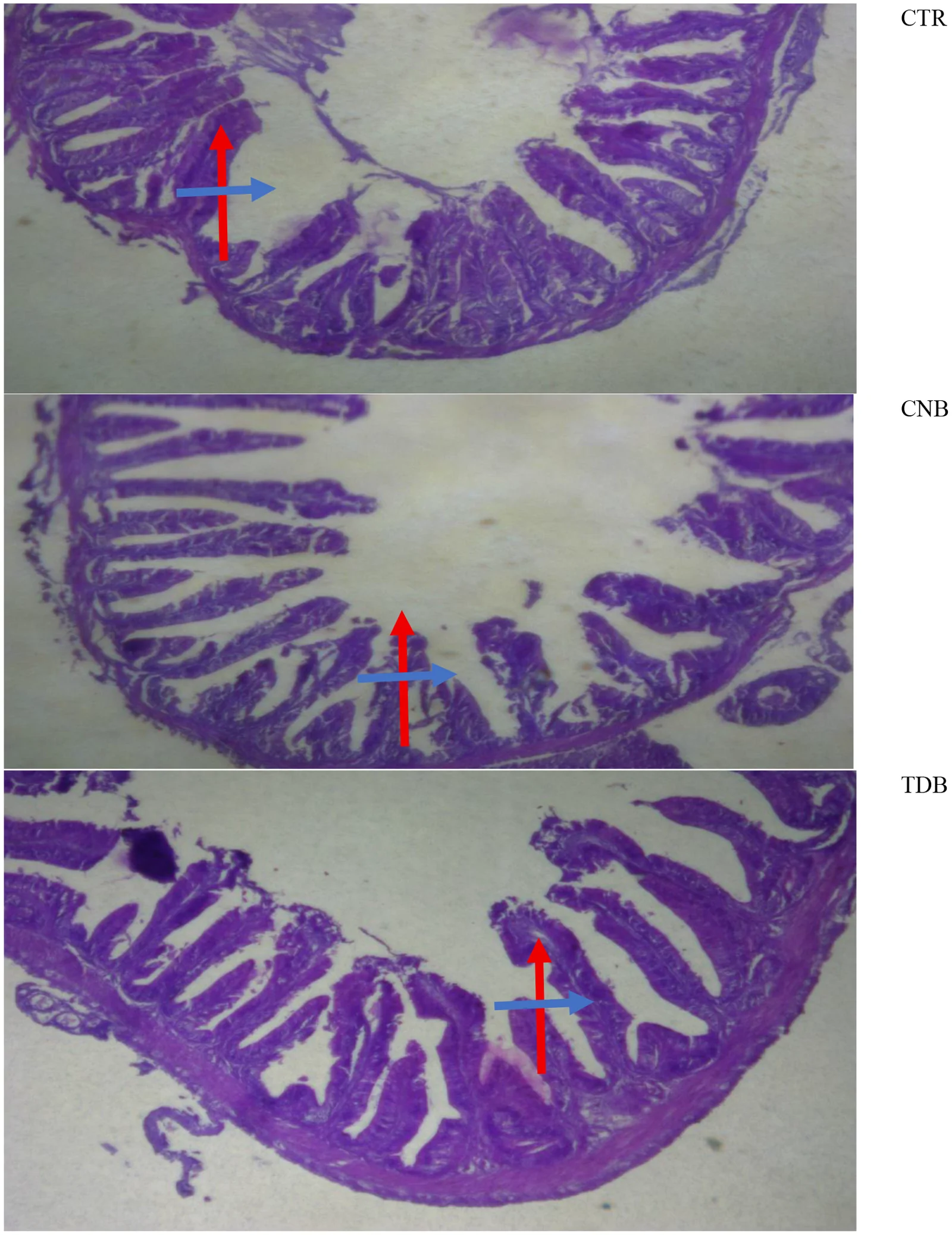
Intestine of Nile tilapia, *Oreochromis niloticus*, Digesta pH, viscosity, and short-chain fatty acids of Nile tilapia raised in RAS for 56 days. Treatments: CTR, no inoculation, standard biofiltration and sulfur pellets as electron donor; CNB, conventional nitrifying biofilter, enriched with *Nitrosomonas* and *Nitrobacter* (1.0 ×10^6^ CFU mL^-1^) at ratio of two to one, respectively and sulfur pellets as electron donor; TDB, *T. denitrificans*-inoculated biofilter, enriched with *T. denitrificans* (1.0 ×10^6^ CFU mL^-1^) and sulfur pellets as electron donor). arrow (red colour) = villus length, arrow (blue colour) villi width (HE ×40, 100) (n=3).

The influence of *T. denitrificans* on gut histomorphometry is presented in **Table 7** and **Figure 3**. The villi height (*P* = 0.001), villi width (*P* = 0.003), cryptal depth (*P* = 0.012), area of absorption (*P* = 0.001), and the villi length:villi width ratio (*P* = 0.001) increased in TDB compared to the control.

**Table 7:** Gut histomorphometry of Nile tilapia cultured in *T. denitrificans* induced RAS for 56 days.

Paramters	CTR	CNB	TDB	PSD	*P*- value
Villi length (cm)	0.32 ^b^	0.48 ^b^	0.83 ^a^	0.014	0.001
Villi width (cm)	0.26 b^a^	0.29 ^b^	0.42 ^a^	0.008	0.003
Crytal depth (cm)	0.16	0.19	0.23	0.002	0.012
Area of absorption (cm^2^)	0.08 ^c^	0.14 ^b^	0.35 ^a^	0.002	<0.001
Villi length:Villi width	1.23 ^c^	1.62 ^b^	1.95 ^a^	0.027	0.001

Means having the same letter in the same row are not significantly different at *P* > 0.05.

CTR, no inoculation, standard biofiltration and sulfur pellets as electron donor; CNB, conventional nitrifying biofilter, enriched with *Nitrosomonas* and *Nitrobacter* (1.0 ×10^6^ CFU mL^-1^) at ratio of two to one, respectively and sulfur pellets as electron donor; TDB, *T. denitrificans*-inoculated biofilter, enriched with *T. denitrificans* (1.0 ×10^6^ CFU mL^-1^) and sulfur pellets as electron donor; PSD, Pooled standard deviation.

### 3.10 Immune responses of Nile tilapia

**Table 8** revealed the non-specific immunity of Nile tilapia. There were significant differences in the immune profiles of the fish (*P* < 0.05). RBA (*P* = 0.001), LYZ (*P* = 0.001), PTA (*P* = 0.002), APP (*P* = 0.014), and ETA (*P* = 0.001) were significantly elevated in fish raised in TDB, while the least were observed in the control CTR group.

**Table 8:** Immune response of Nile tilapia induced with *T. denitrificans* for 56 days.

Parameters	CTR	CNB	TDB	PSD	*P*-value
RBA (mg mL^-1^)	156.8 ^c^	274.2 ^b^	285.5 ^a^	7.865	0.001
LYZ (Ug mL^-1^)	4.23 ^c^	5.70 ^b^	6.53 ^a^	0.642	<0.001
PTA (U mg^-1^ protein)	54.7 ^c^	66.3 ^b^	77.1 ^a^	2.234	0.002
APP (IU L^-1^)	132.8 ^c^	145.8 ^b^	158.4 ^a^	6.07	0.014
ETA (U mg^-1^ protein)	35.2 ^c^	44.5 ^b^	52.5 ^a^	82.5	<0.001

Means having the same letter in the same row are not significantly different at *P* > 0.05.

RBA, Respiratory burst activity; LYZ, Lysozyme; PTA, Protease activity; APP, Alkaline phosphatase; ETA, Esterase activity.

CTR, no inoculation, standard biofiltration and sulfur pellets as electron donor; CNB, conventional nitrifying biofilter, enriched with *Nitrosomonas* and *Nitrobacter* (1.0 ×10^6^ CFU mL^-1^) at ratio of two to one, respectively and sulfur pellets as electron donor); TDB, *T. denitrificans*-inoculated biofilter, enriched with *T. denitrificans* (1.0 ×10^6^ CFU mL^-1^) and sulfur pellets as electron donor), PSD, Pooled standard deviation.

## 4 Discussion

### 4.1 Nitrogen removal, water quality, and microbial abundance

Removal of nitrogen in fish culture systems is critical for optimum fish performance. In this study, raising tilapia in RAS inoculated with TDB resulted in greater removal of nitrogen, specifically TAN, nitrite, and nitrate. This reduction pointed that *T. denitrificans* plays an indirect environmental role and acts as a denitrification (facultative chemolithotrophic sulfur‐oxidizing denitrifier) enhancer due to its ability to oxidize reduced sulfur compounds and consequently reduce nitrate to nitrogen gas. A lower nitrite level observed in the TDB group suggests that *T. denitrificans* stimulated the conversion of nitrite to nitrate. In a similar study, nitrate reduction was reported by Zhang et al. (2023) when water was inoculated with sulfur-driven denitrification bacteria. Also, the decrease in the level of TAN in the TDB group reveals that *T. denitrificans* helps in maintaining the ammonia below the toxic level. *T. denitrificans* reduced the ammonia, nitrite, and nitrate levels in water from the tank used to raise Nile tilapia (Maia et al., 2025) with values (> 79% removal) similar to what was observed in this study. The observed results are explained by the ability of the TDB to actively convert nitrate to nitrogen gas, enhancing nitrification-denitrification coupling and system stability, leading to effective nitrogen removal in tilapia farms and, by implication, indirectly reducing the water usage and exchange. Although denitrification directly converts nitrate to gaseous nitrogen rather than ammonia, the significantly lower ammonia concentration observed in the TDB treatment can be explained by enhanced coupling of nitrification and denitrification within the biofilter. *T. denitrificans* primarily contributes to nitrate reduction, while ammonia removal is mediated principally by nitrifying microorganisms and microbial assimilation. Continuous consumption of nitrate by sulfur-driven denitrification may facilitate sustained upstream nitrification by preventing nitrate accumulation and maintaining a favorable nitrogen-transformation gradient. In addition, colonization of the sulfur-based biofilter by diverse microorganisms may have enhanced microbial assimilation of ammonium (Maia et al., 2025).

Water is crucial to the survival and physiological functions of fish. Since nitrogenous wastes, especially from leftover feed and feces, directly affect water, its management cannot be overemphasized. In the present study, inoculation of *T. denitrificans* (i.e., water from the TDB system) has distinct and clearer water quality parameters, especially the decrease in the TSS and COD. These results point to *T. denitrificans* influencing the nitrogen cycle and broader biogeochemistry, which eventually improved overall water quality. The ability of *T. denitrificans* to reduce nitrate to nitrogen gas helps in eliminating the organic overload and reducing the TSS and COD in the system. The improved nitrogen-related water quality alongside the reductions in TSS and COD is in agreement with the findings of Ma et al. (2024), who reported that inclusion of *T. denitrificans* leads to regulation of inorganic nitrogen and organic loads in RAS.

In relation to general microbial abundance, *T. denitrificans* greatly altered the microbial abundance in the RAS. This modification points to a reassembled microbial abundance in a positive way by promoting the growth of possible beneficial bacteria and suppressing the enteric bacteria in the system. The increase in the TVC in the TDB group credibly indicates successful colonization of *T. denitrificans* in the system. A study showed that autotrophic denitrifiers such as *T. denitrificans* can change local redox, sulfur, and nitrogen fluxes and modify substrates to favor the growth of beneficial bacteria (Huang et al., 2024). In addition, the reduction in TEB in TDB may indicate a direct antagonism, quick colonization by outcompeting, and/or pH modification to exclude the TEB. Autotrophic denitrifiers such as *T. denitrificans* decrease the population of pathogenic or enteric indicator bacteria (Maia et al., 2025). The improved microbial abundance in TDB underscores the importance of the inclusion of *T. denitrificans* as a microbial abundance promoter and inhibitor of pathogens (Luo et al., 2022).

### 4.2 Growth performance and feed utilization

The results of the growth study revealed that TDB greatly improved both growth performance and feed utilization without compromising fish viability. Studies have shown that probiotics and denitrifiers in RAS enhanced the growth of tilapia and feed utilization, especially when water quality is at the same time improved (Mohammed et al., 2025). These reports are concomitant with the present study, where inoculation of *T. denitrificans* caused higher FBW, BDW, and SGR and lower FCR. Furthermore, the improved growth and lowered FCR recorded in TDB aligned with the improved water quality and microbial abundance. This is because reduction in TSS and COD, stable DO, and temperature would reduce chronic sub-lethal stress on fish and, consequently, indirectly enhance feed intake, feed conversion, and, finally, growth. More so, fish will expend less energy on osmoregulation and stress responses and, therefore, use energy for growth. The lower feed intake in TDB may be associated with the substantially improved water quality and reduced environmental stress observed in this treatment. TDB had lower TAN, nitrite and nitrate concentrations, as well as reduced TSS and COD, while dissolved oxygen and temperature remained stable. These conditions may have reduced the energetic costs associated with physiological stress and enabled fish to utilize consumed nutrients more efficiently for tissue growth rather than maintenance and stress responses. Similarly, modification in the microbial abundance tends to increase microbial biomass. This biomass serves as indirect supplementary feed and reduces nitrogen stress in fish. Nile tilapia raised in a *T. denitrificans* biofloc system had improved growth, which is in agreement with the present study (Maia et al., 2025).

### 4.3 Short-chain fatty acids and histomorphometry

Fatty acids play critical roles in gut health management and general metabolic regulation in fish and have become an important tool in fish health management, nutrition, and fish welfare. This study demonstrated that Nile tilapia in the TDB group showed significantly higher levels of acetic and butyric acids when compared to the fish in the control group. Acetate and butyrate help in microbial fermentation, which promotes gut epithelial integrity, serves as an energy substrate for intestinal cells, and evokes immunomodulatory and antioxidant effects (Eissa et al., 2024; Adeshina et al., 2025). The elevated SCFAs recorded in this suggest that *T. denitrificans* indirectly promotes microbial fermentation, and more importantly, insignificant pH or propionic acid levels further explain that the microbial alteration was selective for the beneficial bacteria. Gut histomorphometry of Nile tilapia in TDB-based RAS was greatly improved, though indirect, pointed to an increase in the absorptive and digestive capacity of the intestine of the fish and eventually resulted in higher nutrient absorption and improved growth due to an improved water quality and reduced nitrogen stress. Increased villi height and area of absorption extend the epithelial surface available for nutrient assimilation, while deeper crypts may indicate greater regenerative potential of enterocytes.

### 4.4 Hematological and antioxidant profiles and immune responses

Hematological evaluation is a good indicator of the evaluation of the nutritional status and health status of fish. As shown in this study, TDB significantly influenced the hematological parameters of Nile tilapia. Hematological profiles including PCV, Hb levels, and RBC counts were significantly increased, highlighting the positive influence of TDB on fish health by improving blood profiles. TDB also enhanced the oxygen-carrying capacity and overall vitality of Nile tilapia. To an extent, *T. denitrificans* acted basically via enhancement of water quality by lowering TAN, nitrite, and nitrate, leading to the ameliorated physiological stress. When water quality stress is reduced, erythropoiesis will be normalized, and PCV, Hb, and RBC will be increased, as cleaner water is directly associated with an improved hematological profile in tilapia (Ende et al., 2024). The slight elevation in WBC in this study suggests an uplift in leukocyte production and an indirect immunostimulatory role of *T. denitrificans*. Similar results were reported by Eissa et al. (2024), who reported that elevated hematological indices were recorded in red tilapia (*Oreochromis niloticus × O. mossambicus*) treated with *Bacillus* spp. However, in this study, differential counts were not altered. The implication is that inoculation that improves overall fish performance and water quality seems to influence WBC and may not greatly modify the differential counts except when there is an infectious or inflammatory challenge. Lymphocytes are important components of the adaptive immune system and play key roles in antibody production and cellular immune responses. Therefore, the observed variation in LYM among treatments suggests that the experimental treatments may have influenced the immune competence of the fish. The finding of this study agreed with the findings of Eissa et al. (2024), who reported no significant differences in the differential counts of red tilapia raised in water supplemented with probiotics.

The present study revealed that inoculation of *T. denitrificans* into the RAS markedly influenced the biochemical profiles of Nile tilapia. The increase in TP of fish in the TDB group expressed an enhanced protein metabolism and improved physiological condition. TP is directly linked to nutrient utilization and lowered catabolic stress and has served as an important biomarker of fish health status. The reduction in GLU levels further supports that water quality in TDB-inoculated RAS ameliorates nitrogen-associated stress, as elevation in TAN or nitrite could lead to hyperglycemia in fish (Eissa et al., 2024). Thus, the reduction in GLU implies that *T. denitrificans* indirectly stabilized the metabolic state of the fish. In the same vein, CHO was reduced in the TDB group, which shows the fish were able to metabolize the lipid well. This is consistent with the findings of Abdel-Tawwab et al. (2023). Furthermore, the great reduction in ALT activity, with no major influence on AST and ALP, revealed that TDB inclusion evoked hepatoprotective effects on the fish. ALT is a sensitive biomarker of liver injury and hepatocellular leakage in fish; its reduction implies improved hepatocyte integrity and reduced metabolic load. Similar findings have been documented in Nile tilapia reared under water supplemented with probiotics, where ALT activity was lowered while AST and ALP remained unchanged (Eissa et al., 2024). The stability of AST and ALP in the present study suggests that TDB primarily ameliorated mild hepatocellular stress rather than affecting broader hepatic or biliary enzyme pathways.

As regards antioxidant profiles, fish raised in the TDB-based RAS showed higher antioxidant activity and significantly lower MDA. The reduction in the MDA in TDB fish indicates reduced lipid peroxidation and overall oxidative damage. In addition, the results further suggest that TDB-based RAS had a reduced oxidative stress encumbrance in the fish through the help of microbial metabolites and modulation of antioxidant pathways, as well as improved water quality. The reduction in nitrogenous load by *T. denitrificans* could lower ROS build-up and thus improve antioxidant status in largemouth bass (*Micropterus nigricans*) (Xia et al., 2024) and Nile tilapia. Similar findings were reported in Nile tilapia (Shija et al., 2024). Although many studies applied bacteria as feed supplements, whose primary mode of action is through gut mediation and absorption. In this study, the results were better because *T. denitrificans* plays multiple roles, which include gut-mediator agents and water bioaugmentation by improving water quality via denitrification rather than gut colonization. These help Nile tilapia to acquire better health status and reduce external chemical stressors.

The results of this study demonstrate that *T. denitrificans* inoculation greatly evoked non-specific immune responses of Nile tilapia compared with the control group. Assessment of non-specific immune activities such as RBA and LYZ has become a common tool in evaluation of fish immunity (Paray et al., 2025). LYZ is a component of the innate immune system that plays a crucial role in defense against foreign invasion due to its opsonization properties (Kamble et al., 2024). Studies have shown the stimulatory roles of bacteria on RBA and LYZ in fish. In this study, a significant improvement in both LYZ and RBA levels was recorded in fish in TDB, as compared to those in the CTR group. An increase in lysozyme and respiratory burst activities may be due to an improved water quality, which facilitates immunostimulatory effects of T. *denitrificans*. A rapid increase in respiratory burst activities further intensifies the activity of lysozyme. These findings suggest that TDB supplementation can bolster innate immune functions in Nile tilapia via reduced nitrogen stress. This study agrees with what was observed in Nile tilapia (Eissa et al., 2024) and largemouth bass (Xia et al., 2024) raised in a bacteria-inoculated system, which demonstrated a higher RBA and LYZ than fish in a control system. The marked increase in non-specific immune markers in TDB-treated fish indicates that bioaugmentation with denitrifying bacteria can perform multifunctionally by improving water quality and simultaneously evoking immunity. These improvements are valuable especially in RAS, where fish are constantly challenged by stress and opportunistic pathogens if not properly managed. This study is the first to demonstrate the integration of *T. denitrificans* into RAS for Nile tilapia production and establishes a link between a simultaneous reduction of ammonia, nitrite, and nitrate as well as organic load and improvement in fish growth, immunity, antioxidant status, and SCFAs, indicating a dual functional role of *T. denitrificans*. This integrated approach provides a novel, sustainable strategy for improving productivity and immune modulation in Nile tilapia.

## 5 Conclusion

This study demonstrated that inoculating *Thiobacillus denitrificans* in a RAS significantly improved water quality, fish performance, and the health of Nile tilapia. More interestingly, *T. denitrificans* effectively reduced TAN, nitrite, nitrate, TSS, and COD without negative effects on the DO. Consequently, it translates into enhanced growth, with higher body weight gain, and specific growth rate, alongside a lower feed conversion ratio, without compromising survival. Also, fish reared in the inoculated RAS showed improved growth, hematological and antioxidant capacity, and immune profiles in Nile tilapia.

## Data Availability

The original contributions presented in the study are included in the article/**Supplementary Material**, further inquiries can be directed to the corresponding author/s.
